# IGF-1 Interacted With Obesity in Prognosis Prediction in HER2-Positive Breast Cancer Patients

**DOI:** 10.3389/fonc.2020.00550

**Published:** 2020-04-24

**Authors:** Yiwei Tong, Jiayi Wu, Ou Huang, Jianrong He, Li Zhu, Weiguo Chen, Yafen Li, Xiaosong Chen, Kunwei Shen

**Affiliations:** Comprehensive Breast Health Center, Ruijin Hospital, Shanghai Jiao Tong University School of Medicine, Shanghai, China

**Keywords:** breast cancer, HER2-positive, IGF-1, metabolic syndrome, disease outcome

## Abstract

**Purpose:** Dysmetabolism and high circulating insulin-like growth factor 1 (IGF-1) would increase breast cancer risk, but its association with survival in HER2+ breast cancer patients has not been well-studied. Herein, we aim to evaluate the prognostic value of IGF-1 and metabolic abnormalities in HER2+ population.

**Patients and Methods:** HER2+ breast cancer patients treated in Ruijin Hospital between November 2012 and June 2017 were retrospectively analyzed. Median value of circulating IGF-1 was adopted to classify low or high IGF-1 group. Metabolic syndrome (MetS) was defined using AHA/NHLBI criteria. Overweight was defined by body mass index (BMI) ≥ 24.0 kg/m^2^ in Chinese population.

**Results:** Overall, 679 patients were included and 209 had synchronous MetS. High IGF-1 level was more common in pre/peri-menopausal women (*P* < 0.001) and high IGFBP-3 patients (*P* < 0.001). After a median follow-up of 36 months, 52 patients had disease recurrences. IGF-1 level was not associated with recurrence-free survival (RFS, *P* = 0.620) in the whole population. However, exploratory subgroup analysis found that BMI and IGF-1 interacted in predicting RFS (*P* = 0.009). For non-overweight patients, high IGF-1 showed a superior 4-years RFS (91.1 vs. 85.0%; HR 0.53, 95% CI 0.27–1.00, *P* = 0.049) compared with patients with low IGF-1 level. In contrast, for overweight patients, high IGF-1 was associated with an impaired 4-years RFS (88.3 vs. 95.7%, HR 3.20, 95% CI 1.00–10.21, *P* = 0.038). Furthermore, high IGF-1 level was independently associated with better OS in the whole (HR 0.26, 95% CI 0.08–0.82, *P* = 0.044) as well as non-overweight population (HR 0.15, 95% CI 0.03–0.68, *P* = 0.005).

**Conclusions:** IGF-1 level was not associated with RFS in HER2+ breast cancer patients. However, IGF-1 and BMI had significant interaction in disease outcome prediction in HER2+ patients. High IGF-1 was protective in non-overweight patients, but risk factor for those overweight, which deserves further evaluation.

## Introduction

Breast cancer (BC) is the most common malignancy in women worldwide. According to the latest world cancer statistics, there are an estimated 2.1 million newly diagnosed female BC cases in 2018, accounting for 11.6% of all tumor events and 25% of female tumor events (1). About 15–20% BC cases see overexpression of human epidermal growth factor receptor-2 (HER2), which confers more aggressive tumor biological behavior and poorer disease outcomes (2). Tumor size, node involvement, histological grade, hormone receptor status, proliferation index, HER2-enrichment intrinsic subtype and anti-HER2 therapy application are identified prognostic factors for HER2-positive population (3).

Metabolic syndrome (MetS) is a group of pathophysiological disturbances, including central obesity, insulin resistance, dyslipidemia, and elevated blood pressure. Evidence showed that adult women with MetS had a significantly increased BC risk and a worse clinical outcome (4–8). Potentially suggested mechanisms include the regulation of chronic inflammation, adipose-derived cytokines, and aromatase activity (9, 10). It is also proposed that insulin resistance and insulin-like growth factor 1 (IGF-1) system play a critical role in the association between MetS and BC risk.

The IGF-1 system, including IGF-1, IGF-binding proteins (IGFBPs) and IGF-1 receptor (IGF-1R), is an essential part in cellular proliferation and breast tissue development (11). In both normal and malignant breast tissues, IGF-1 is mostly secreted by stromal cells, while its ligand, IGF-1R, is mainly produced by mammary epithelium (11). The IGF-1 and IGF-1R binding stimulates subsequent phosphatidylinositol 3-kinase (PI3K) and mitogen activated protein kinase (MAPK) pathway activation, two major signaling cascades for cell proliferation. Meanwhile, a family of IGF binding proteins (IGFBP-1 to IGFBP-6) can compete with IGF-1R to bind circulating IGF-1 with high affinity and specificity, thus moderating IGF bioavailability and half-life (11). *In vivo*, majority of the IGF-1 molecules are combined to IGFBP-3 to form a ternary complex in serum.

IGF-1 signaling is involved in 87% invasive BC patients (12). It is hypothesized that potential crosstalk exists between IGF-1 pathway and epidermal growth factor receptor family. Increased IGF signaling tends to support the progression of BC and to promote resistance to established therapies (12), causing decreased BC-specific survival (13) and increased all-cause mortality (14) in HER2-positive subtype. As shown by Camirand et al. co-blocking HER2 and IGF-1R inhibits the growth of HER2-overexpressing BC cells (15). Our previous study in HER2-positive population receiving neoadjuvant treatment (NAT) revealed that pre- and post-NAT IGF-1 expression can reflect NAT efficacy, and that lower IGF-1 expression before the initiation of NAT is an independent predictor for pathologic complete remission (pCR) (16). However, IGF-1 level was shown to be unrelated to overall survival (OS) or disease-free survival (DFS).

Overall, the association between IGF-1, metabolic variables, and clinical outcomes in HER2-amplified early breast cancer population hasn't been well-understood. The objective of the current study is thus to investigate the prognostic value of IGF-1 and MetS components in HER2-positive BC patients.

## Patients and Methods

### Patient Population

BC patients who received operation between November 2012 and June 2017 in Comprehensive Breast Health Center, Ruijin Hospital, Shanghai Jiao Tong University School of Medicine, Shanghai, China were retrospectively analyzed. Eligibility criteria included: (1) female gender; (2) HER2-positive disease conforming to the 2018 ASCO/CAP guidelines, described as immunohistochemistry HER2 3+, immunohistochemistry HER2 2+ and fluorescence *in situ* hybridization *HER2* amplified (*HER2*/*CEP17* ratio ≥ 2.0 and *HER2* copy number ≥ 4.0, *HER2*/*CEP*17 ratio <2.0 and *HER2* copy number ≥ 6.0); (3) available metabolic variables and anthropometrics including blood pressure, glucose and lipid metabolism parameters; (4) complete follow-up information. Patients with complications such as hypertension, diabetes, obesity, etc. can be included in the study. Patients receiving NAT or diagnosed with *de novo* stage IV disease were excluded. This approach was approved by the independent Ethical Committees of Ruijin Hospital, Shanghai Jiao Tong University School of Medicine. All procedures involving human participants were in accordance with the ethical standards of national research committee and with the 1964 Helsinki declaration and its later amendments or comparable ethical standards.

### Data Collection

Patient clinical data were retrieved from Shanghai Jiao Tong University Breast Cancer Database (SJTU-BCDB). Tumor histopathologic examination was performed by two independent experienced pathologists in the Department of Pathology, Ruijin Hospital, Shanghai Jiao Tong University School of Medicine. HER2-positivity was confirmed according to the 2018 ASCO/CAP guidelines as described above. Methods and positivity definition adopted for immunohistochemical evaluation of other tumor biomarkers were as described in our previous reports (17).

The measurement of metabolic parameters was performed in the Department of Clinical Laboratory, Ruijin Hospital using peripheral blood samples collected by trained nurses before surgery. Fasting glucose and lipid metabolism parameters including triglycerides, total cholesterol (TC), high-density lipoprotein cholesterol (HDL-C), and low-density lipoprotein cholesterol (LDL-C), were assessed using Beckman Coulter-AU5800 (Beckman Coulter, Inc., Atlanta, GA, USA). IGF-1 and IGFBP-3 were tested by chemiluminescent immunoassay using IMMULITE 2000 system IMMULITE 2000 system (Siemens AG, Munich, Germany). Serum insulin and C-peptide were tested by electrochemiluminescence immunoassay on Cobas E601 analyzers (Hoffman-La Roche Ltd, Basel, Switzerland).

American Heart Association/National Heart, Lung, and Blood Institute (AHA/NHLBI) guideline was applied to define MetS in the current study after being modified according to the Chinese population (18). Chinese body mass index (BMI) cutoff of ≥ 24.0 kg/m^2^ for overweight was employed to define central obesity according to previous studies (19–21).

### Follow-Up

Patient follow-up was carried out by BC-specialized nurses in our center. Recurrence-free survival (RFS) was calculated from the date of the surgery to the first proven recurrent event including ipsilateral and local/regional recurrence, distant metastasis in any site, and death of any cause. Diagnosis of disease recurrence was based on radiographic images and/or pathologic biopsies when possible. OS was calculated till death of any cause. The last follow-up was completed in May 2019.

### Statistical Analysis

Median values for metabolic parameters were adopted as cutoff to turn numerical variables into categorical variables. Chi-square test and multivariate logistic regression were applied to clarify the distribution of clinical variables regarding the IGF-1 expression. One-way ANOVA test was applied to compare the distribution of IGF axis related variables by recurrent status. Kaplan–Meier curve and Cox regression model were employed for survival analysis. Subgroup analysis was conducted with the stratified Mantel-Haenszel model to estimate the potential interaction of biomarkers. All statistical procedures were realized in IBM SPSS statistics software version 23 (IBM Corporation, Armonk, NY, USA) and GraphPad Prism version 7.0 (GraphPad Software, CA, USA). Two-sided *P* < 0.05 were considered statistically significant.

## Results

### Baseline Characteristics and Clinical Treatment

In all, 679 HER2-positive BC patients were included. Patient baseline characteristics are summarized in Table 1. The mean age was 53.04 ± 10.59 (range: 23–95) years. A total of 529 (77.91%) patients received mastectomy, and 58.76% received sentinel lymph node biopsy (SLNB). 94.55% of the patients were diagnosed with invasive ductal carcinoma (IDC), while others had invasive lobular carcinoma, mucinous carcinoma, or mixed carcinoma, etc. Grade III tumors were found in 65.98% (448/679) of the patients. Tumor size was no more than 2.0 cm in 315 patients. Of the enrolled population, 420 were node-negative, while 55.96 and 72.61% had ER or PR negative disease. Median Ki67 expression was 30% in the study population. HER2-overexpressed and Luminal-B HER2-positive diseases were found in 55.96% and 44.04% cases. In the adjuvant setting, 606, 355, 544, and 296 patients received chemotherapy, radiotherapy, targeted therapy, and endocrine therapy, respectively.

**Table 1:** Baseline clinical characteristics and clinical treatment of HER2-positive breast cancer patients.

**Characteristics**	**Total *N* = 679**	**Low IGF-1 *N* = 337 (%)**	**High IGF-1 *N* = 342 (%)**	***P*-value**
**Age at Diagnosis, Years**				<0.001
<50	244	88 (26.11)	156 (45.61)	
≥ 50	435	249 (73.89)	186 (54.39)	
**Menopausal Status**				<0.001
Pre/Peri-menopausal	285	99 (29.38)	186 (54.39)	
Post-menopausal	394	238 (70.62)	156 (45.61)	
**Histologic Type**				0.003
IDC	642	310 (91.99)	332 (97.08)	
Non-IDC	37	27 (8.01)	10 (2.92)	
**Histological Grade**				0.451
I-II	231	110 (32.64)	121 (35.38)	
III	448	227 (67.36)	221 (64.62)	
**Tumor Size, cm**				0.411
≤ 2.0	315	151 (44.81)	164 (47.95)	
> 2.0	364	186 (55.19)	178 (52.05)	
**Node Status**				0.347
Positive	256	133 (39.47)	123 (35.96)	
Negative	420	201 (59.64)	219 (64.04)	
N/A	3	3 (0.89)	0 (0.00)	
**ER Status**				0.322
Positive	299	142 (42.14)	157 (45.91)	
Negative	380	195 (57.86)	185 (54.09)	
**PR Status**				0.109
Positive	186	83 (24.63)	103 (30.12)	
Negative	493	254 (75.37)	239 (69.88)	
**Ki67, %**				0.713
<30	212	103 (30.56)	109 (31.87)	
≥ 30	467	234 (69.44)	233 (68.13)	
**Molecular Subtype**				0.322
HER2-amplified	380	195 (57.86)	185 (54.09)	
Luminal B-like	299	142 (42.14)	157 (45.91)	
**Adjuvant Chemotherapy**				0.153
Yes	606	295 (87.54)	311 (90.94)	
No	73	42 (12.46)	31 (9.06)	
**Adjuvant Radiotherapy**				0.208
Yes	355	168 (49.85)	187 (54.68)	
No	324	169 (50.15)	155 (45.32)	
**Adjuvant Targeted Therapy**				0.021
Yes	544	258 (76.56)	286 (83.63)	
No	135	79 (23.44)	56 (16.37)	
**Adjuvant Endocrine Therapy**				0.285
Yes	296	140 (41.54)	156 (45.61)	
No	383	197 (58.47)	186 (54.39)	

*HER2, human epidermal growth factor receptor-2; IGF-1, insulin-like growth factor-1; IDC, invasive ductal carcinoma; N/A, not available; ER, estrogen receptor; PR, progesterone receptor*.

The metabolic variables and anthropometrics are summarized in Table 2. The most common dysmetabolism was hypertension (51.25%), followed by reduced HDL-C (44.77%), overweight (34.76%), elevated fasting glucose (24.59%), and elevated triglycerides (20.62%). There were 209 patients with synchronous MetS at the time of diagnosis, with an average MetS component number of 1.76 ± 1.40.

**Table 2:** Metabolic variables in HER2-positive breast cancer patients.

**Characteristics**	**Total *N* = 679**	**Low IGF-1 *N* = 337 (%)**	**High IGF-1 *N* = 342 (%)**	***P*-value**
**BMI, kg/m**^**2**^				0.080
<24.0	443	209 (62.02)	234 (68.42)	
≥ 24.0	236	128 (37.98)	108 (31.58)	
**Elevated Blood Pressure**				<0.001
Yes	348	197 (58.46)	151 (44.15)	
No	331	140 (41.54)	191 (55.85)	
**Fasting Glucose, mmol/L**				0.463
<5.60	512	250 (74.18)	262 (76.61)	
≥ 5.60	167	87 (25.82)	80 (23.39)	
**Triglycerides, mmol/L**				0.922
<1.70	539	267 (79.23)	272 (79.53)	
≥ 1.70	140	70 (20.77)	70 (20.47)	
**HDL-C, mmol/L**				0.429
<1.30	304	156 (46.29)	148 (43.27)	
≥ 1.30	375	181 (53.71)	194 (56.73)	
**IGFBP-3**, μg/mL*				<0.001
<4.06	250	176 (69.57)	74 (29.84)	
≥ 4.06	251	77 (30.43)	174 (70.16)	
**Insulin**, **μIU/mL**				<0.001
<7.71	339	194 (57.57)	145 (42.40)	
≥ 7.71	340	143 (42.43)	197 (57.60)	
**C-peptide**, **μg/L**				0.001
<1.93	338	189 (56.08)	149 (43.57)	
≥ 1.93	341	148 (43.92)	193 (56.43)	
**MetS at Diagnosis**				0.381
Yes	209	109 (32.34)	100 (29.24)	
No	470	228 (67.66)	242 (70.76)	
**No. of MetS Components**				0.033
0	145	58 (17.21)	87 (25.44)	
1–2	325	170 (50.45)	155 (45.32)	
≥ 3	209	109 (32.34)	100 (29.24)	

* *There were 178 missing values for IGFBP-3, 84 in the low IGF-1 group and 94 in the high IGF-1 group. HER2, human epidermal growth factor receptor-2; IGF-1, insulin-like growth factor-1; BMI, body mass index; HDL-C, high-density lipoprotein-cholesterol; IGFBP-3, insulin-like growth factor binding protein-3; MetS, metabolic syndrome; No, number*.

### Clinico-Pathologic Features, Metabolic Variables and IGF-1 Expression

The median IGF-1 of the participants, 160.00 ng/mL, was adopted to define low or high IGF-1 expression. No significant difference was present regarding histological grade, tumor size, node status, ER, PR, Ki67 statuses and molecular subtype between two groups by univariate analysis (all *P* > 0.05; Table 1). Age (*P* < 0.001), menopausal status (*P* < 0.001), and histologic type (*P* = 0.003) were statistically differently distributed. Regarding metabolic parameters, BMI, fasting glucose, triglycerides, HDL-C, and MetS distributed equally between IGF-1 high and low groups as shown in Table 2 (all *P* > 0.05), while the distribution of blood pressure (*P* < 0.001), IGFBP-3 (*P* < 0.001), insulin (*P* < 0.001), C-peptide (*P* = 0.001), and the number of MetS components (*P* = 0.033) significantly differed by IGF-1expression.

A multinomial logistic regression showed that the overall distribution of menopausal status and IGFBP-3 level was substantially associated with IGF-1 expression (Table 3). High IGF-1 level was more common in pre/peri-menopausal women (odds ratio [OR] 4.00, 95% confidence interval [CI] 2.08–7.69, *P* < 0.001) and high IGFBP-3 patients (OR 5.48, 95% CI 3.61–8.31, *P* < 0.001).

**Table 3:** Multivariate logistic regression analysis of factors associated with IGF-1 level in HER2-positive breast cancer patients.

**Characteristics**	**OR**	**95% CI**	***P*-value**
Age, years (≥ 50 vs. <50)	1.04	0.53–2.05	0.905
Menstruation (Pre/Peri vs. Post-menopausal)	4.00	2.08–7.69	<0.001
Histologic type (Non IDC vs. IDC)	0.45	0.18–1.15	0.094
BMI, kg/m^2^ (≥ 24.0 vs. <24.0)	0.95	0.55–1.63	0.843
IGFBP-3, μg/mL* (≥ 4.06 vs. <4.06)	5.48	3.61–8.31	<0.001
Insulin, μIU/mL (≥ 7.71 vs. <7.71)	1.64	0.95–2.83	0.074
C-peptide, μg/L (≥ 1.93 vs. <1.93)	1.54	0.87–2.75	0.140
No. of MetS components (1–2 vs. 0)	0.70	0.40–1.25	0.229
No. of MetS components (≥ 3 vs. 0)	0.57	0.26–1.23	0.153

* *There were 178 missing values for IGFBP-3. IGF-1, insulin-like growth factor-1; HER2, human epidermal growth factor receptor-2; OR, odds ratio; CI, confidence interval; IDC, invasive ductal carcinoma; BMI, body mass index; IGFBP-3, insulin-like growth factor binding protein-3; No, number; MetS, metabolic syndrome*.

### Distribution of MetS-Related Variables by Recurrence Status

After a median follow-up of 36.00 (range 4.20–74.93) months, 52 out of 679 patients had disease recurrence, including 13 local recurrences, 19 distant metastases, five contralateral breast cancers, and 15 deaths. MetS-related variables, including BMI, blood pressure, glucose, lipid metabolism parameters, were compared by recurrence status (Table S1). Continuous numeric BMI value, fasting glucose, IGF-1, IGFBP-3, insulin, C-peptide, triglycerides, TC, HDL-C, LDL-C, and the number of MetS components were not different between the two groups. Moreover, IGF-1/IGFBP-3 ratio was much higher in the recurrent patients compared with patients without recurrence (45.14 vs. 40.53, *P* = 0.030). Recurrent patients also had elevated C-peptide level than those without (2.24, vs. 2.04, *P* = 0.045). Furthermore, these two groups of patients showed similar proportion of overweight, elevated blood pressure, elevated fasting glucose, higher level of IGF-1, IGFBP-3, insulin, C-peptide, elevated triglycerides, reduced HDL-C, and MetS (all *P* > 0.05).

### Association Between IGF-1 and RFS in HER2-Positive Breast Cancer

**Figure 1:**
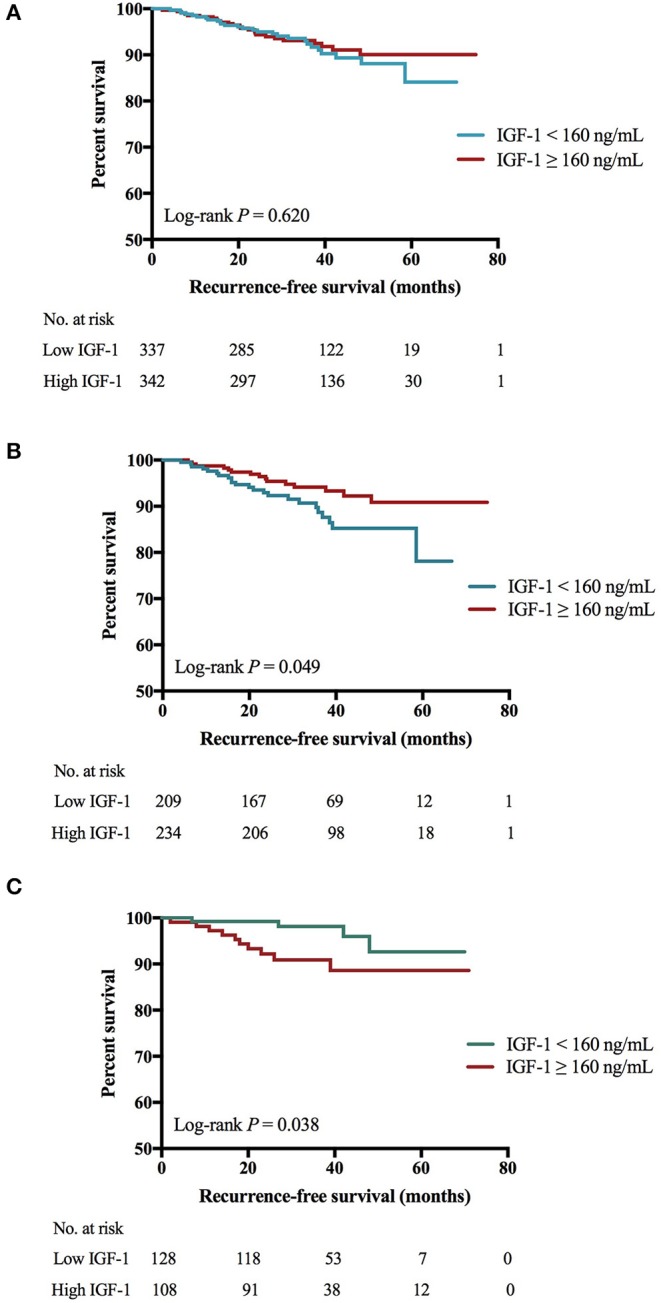
Impact of IGF-1 on RFS among HER2-positive patients. **(A)** There was no significant RFS difference between low and high IGF-1 groups (4-years RFS 89.4 vs. 91.0%, Log-rank *P* = 0.620). **(B)** For patients of BMI <24.0 kg/m^2^, those with higher expression of IGF-1 had superior RFS than those with lower IGF-1 (4-years RFS 91.1 vs. 85.0%, Log-rank *P* = 0.049). **(C)** For patients of BMI ≥ 24.0 kg/m^2^, those with lower expression of IGF-1 had superior RFS than those with higher IGF-1 (4-years RFS 95.7 vs. 88.3%, Log-rank *P* = 0.038). IGF-1, insulin-like growth factor-1; RFS, recurrence-free survival; HER2, human epidermal growth factor receptor 2; BMI, body mass index; No, number.

There was no significant difference of 4-years RFS between low and high IGF-1 groups (89.4 vs. 91.0%, *P* = 0.620; Figure 1A). The 4-years RFS was 90.9 and 87.9% in patients treated with targeted therapy or not (*P* = 0.402).

**Figure 2:**
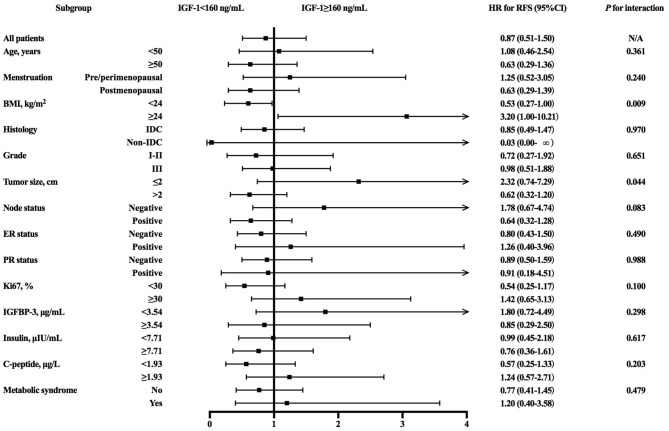
Forest plots and interaction analysis for RFS in HER2-positive breast cancer patients with different level of IGF-1. RFS, recurrence-free survival; HER2, human epidermal growth factor receptor 2; IGF-1, insulin-like growth factor-1; HR, hazard ratio; CI, confidence interval; BMI, body mass index; IDC, invasive ductal carcinoma; ER, estrogen receptor; HR, hormonal receptor; IGFBP-3, insulin-like growth factor binding protein-3.

Further subgroup analysis revealed an interaction between IGF-1 and BMI (*P* for interaction = 0.009; Figure 2) or tumor size (*P* for interaction = 0.044) in predicting RFS. High IGF-1 level was associated with better RFS in the non-overweight patients (4-years RFS 91.1 vs. 85.0%, *P* = 0.049; Figure 1B; hazard ratio [HR] 0.53, 95% CI 0.27–1.00), but with worse outcome in the overweight ones (4-years RFS 88.3 vs. 95.7%, *P* = 0.038; Figure 1C; HR 3.20, 95% CI 1.00–10.21, *P* for interaction = 0.009).

When subdivided on the basis of molecular subtype, IGF-1 expression didn't interact with obesity in RFS prediction. For both overweight or non-overweight Luminal B HER2-positive patients, RFS was similar between those with different IGF-1 levels (Figures S1A,B). For both overweight or non-overweight HER2-overexpressed patients, no significant difference regarding RFS was observed between low or high IGF-1 expression groups, either (Figures S1C,D). We further investigated the relationship between HER2 targeted therapy efficacy and IGF-1 level, interacted with obesity (Figure S2), and found that only in patients with BMI <24.0 kg/m^2^ and receiving adjuvant targeted therapy, those with higher expression of IGF-1 had a tendency of superior RFS than those with lower IGF-1 (Log-rank *P* = 0.053; Figure S2A). In other subgroups, two IGF-1 categories had similar RFS regardless of BMI and targeted therapy use (Figures S2C,E,G).

Univariate and multivariate analyses demonstrated that younger age (*P* = 0.028; Tables S2, S3), lymph node involvement (*P* < 0.001), ER negativity (*P* = 0.002), and PR negativity (*P* < 0.001) were significantly associated with worse RFS in the whole population. IGF-1 status was not independently related with RFS in the whole or non-overweight patients (Table S3, Table 4). However, in overweight patients, age (*P* = 0.015), tumor size (*P* = 0.020), and IGF-1 level (*P* = 0.038) are correlated with RFS in univariate models. Further multivariate analysis identified elder age as a prognostic factor for better RFS (HR 0.27, 95% CI 0.09–0.77, *P* = 0.015; Table 4). High IGF-1 level was significantly inversely associated with RFS in overweight patients (HR 3.65, 95% CI 1.62–8.74, *P* = 0.045).

**Table 4:** Multivariate analysis of prognostic factors affecting RFS in HER2-positive breast cancer patients by BMI status.

**Predictors**	**Non-overweight**			**Overweight**		
	**HR**	**95% CI**	***P***	**HR**	**95% CI**	***P***
Age, years (≥ 50 vs. <50)	0.75	0.39–1.45	0.393	0.27	0.09–0.77	0.015
Tumor size, cm (≥ 2.0 vs. <2.0)	1.21	0.61–2.43	0.585	4.28	0.95–19.23	0.058
Node status (Positive vs. Negative)	5.10	2.53–10.29	<0.001	0.99	0.33–2.92	0.983
ER status (Positive vs. Negative)	0.64	0.25–1.66	0.359	0.35	0.33–2.92	0.317
PR status (Positive vs. Negative)	0.06	0.01–0.45	0.006	3.21	0.37–28.06	0.292
IGF-1, ng/mL (≥ 160 vs. <160)	0.63	0.33–1.21	0.166	3.65	1.62–8.74	0.045
Radiotherapy (Yes vs. No)	0.94	0.38–2.35	0.902	1.12	0.37–3.35	0.845
Endocrine therapy (Yes vs. No)	0.58	0.23–1.50	0.261	1.23	0.07–22.54	0.891

*RFS, recurrence-free survival; HER2, human epidermal growth factor receptor-2; BMI, body mass index; HR, hazard ratio; CI, confidence interval; ER, estrogen receptor; PR, progesterone receptor; IGF-1, insulin-like growth factor 1*.

### Impact of IGF-1 on OS in HER2-Positive Breast Cancer

**Figure 3:**
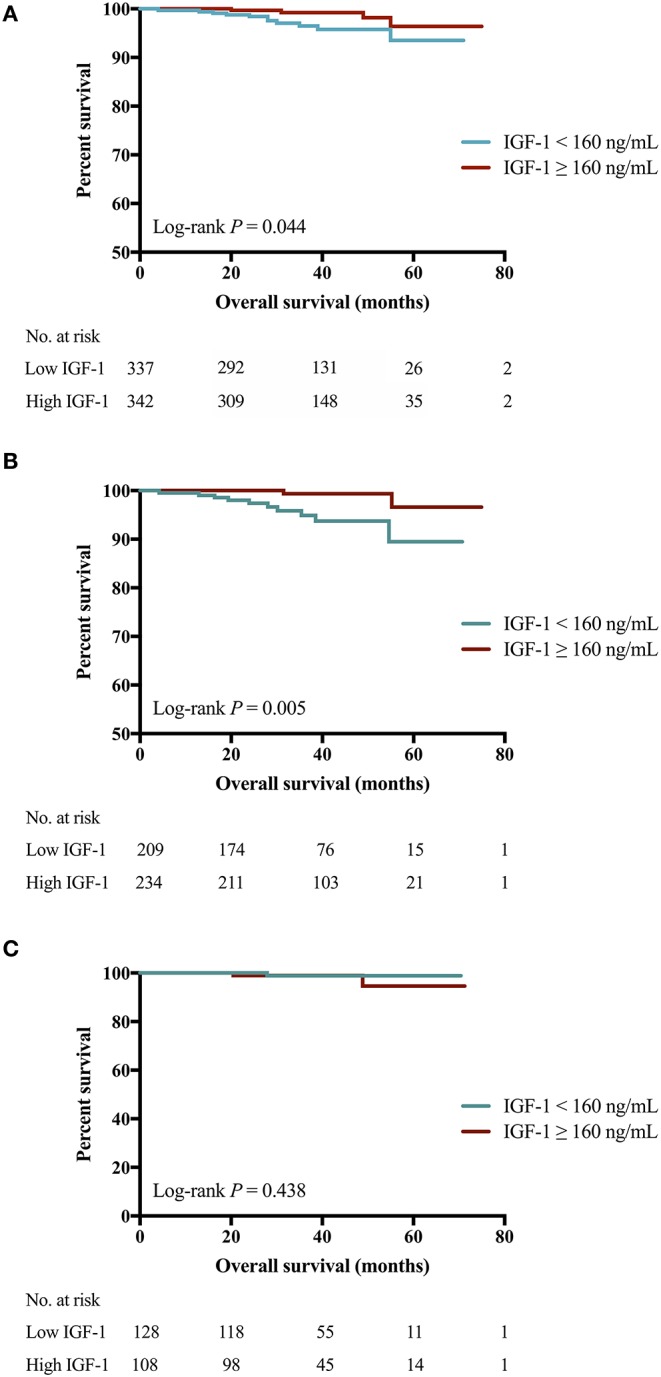
Impact of IGF-1 on OS among HER2-positive patients. **(A)** High IGF-1 group had superior OS than low IGF-1 group (4-years RFS 99.2 vs. 95.8%, Log-rank *P* = 0.044). **(B)** For patients of BMI <24.0 kg/m^2^, those with higher expression of IGF-1 had superior OS than those with lower IGF-1 (4-years OS 99.4 vs. 93.7%, Log-rank *P* = 0.005). **(C)** For patients of BMI ≥ 24.0 kg/m^2^, similar OS was found in those with lower or higher IGF-1 (4-years OS 98.9 vs. 98.7%, Log-rank *P* = 0.438). IGF-1, insulin-like growth factor-1; OS, overall survival; HER2, human epidermal growth factor receptor 2; BMI, body mass index; No, number.

The 4-years OS was 97.7 and 96.7% in patients who received targeted therapy or not (*P* = 0.149). Better 4-years OS was observed in the high IGF-1 group compared to the low IGF-1 group (99.2 vs. 95.8%, *P* = 0.044, Figure 3A). Subgroup analysis showed a modest but insignificant interaction of IGF-1 and BMI in predicting OS (*P* for interaction = 0.054; Figure S3). High IGF-1 level was associated with improved OS in non-overweight patients (4-years OS 99.4 vs. 93.7%, *P* = 0.005; Figure 3B; HR 0.15, 95% CI 0.03–0.68), but not in overweight ones (4-years OS 98.7 vs. 98.9%, *P* = 0.438; Figure 3C; HR 2.51, 95% CI 0.23–27.63, *P* for interaction = 0.054).

For overweight or non-overweight Luminal B HER2-positive patients, IGF-1 expression didn't have significant impact on OS (Figures S4A,B). However, for lean patients with HER2-overexpressed disease, high IGF-1 was significantly associated with better OS (*P* = 0.020, Figure S4C), while such significant no longer persisted for overweight, HER2-overexpressed patients (*P* = 0.317, Figure S4D). Further investigation of the relationship between HER2 targeted therapy efficacy and IGF-1 level, interacted with obesity (Figure S2) showed that only in patients with BMI <24.0 kg/m^2^ and receiving adjuvant targeted therapy, those with higher expression of IGF-1 had a significant superior OS than those with lower IGF-1 (Log-rank *P* < 0.001; Figure S2B). In other subgroups, two IGF-1 categories had similar OS regardless of BMI and targeted therapy use (Figures S2D,F,H).

Both univariate and multivariable analyses found that high IGF-1 (HR 0.26, 95% CI 0.08–0.82, *P* = 0.022; Tables S3, S4), age ≥50 years (*P* = 0.036) and node negativity (*P* = 0.003) were significantly associated with better OS in the whole population. IGF-1 status was not independently related with OS in overweight patients (Table S5). Whereas, regarding non-overweight patients, IGF-1 level (*P* = 0.005), tumor size (*P* = 0.040), and nodal status (*P* < 0.001) were correlated with OS in univariate models. High IGF-1 level was independently related to improved OS (HR 0.15, 95% CI 0.03–0.71, *P* = 0.016; Table S5).

## Discussion

In current study, including 679 HER2-positive patients, we found that menopausal status and IGFBP-3 level were associated with circulating IGF-1 expression level. Among metabolic variables, IGF-1/IGFBP-3 ratio and circulating C-peptide level were significantly higher in the recurrent patients than in the non-recurrent ones. After a median follow-up of 36.00 months, we found a substantial interaction between IGF-1 and BMI on RFS (*P* for interaction = 0.009) and a tendency of interaction of these two factors on OS (*P* for interaction = 0.054). Regarding RFS, high IGF-1 was protective for non-overweight patients, but deleterious for overweight ones. To our knowledge, this is the largest study to evaluate the association between IGF-1 and the survival in HER2-positive BC population, and the first to demonstrate a significant interaction between IGF-1 and BMI.

The management of HER2-amplified breast tumors remained challenging in clinical practice due to its relatively aggressive biological behavior and worse prognosis. Apart from well-established prognostic factors like tumor size, node involvement, grade, hormone receptor status, proliferation index, HER2-enrichment intrinsic subtype and use of anti-HER2 therapy (3), efforts were continuously made to identify reversible and modifiable variables to predict treatment efficacy and disease outcomes. Previous studies indicated the existence of a crosstalk between IGF-1 system and HER2 pathway. Activation of the IGF system was suggested to promote cancer progression, invasion, and treatment resistance (22). Recent meta-analysis demonstrated that higher circulating IGF-1 was significantly associated with increased BC risk (23). Preclinical evidence suggested that a high IGF-1 environment could induce metastases by activating AKT, recruiting NF-κB, and subsequently increasing BC cell proliferation (24). Researches in HER2-positive BC cell lines found that dual blockade of HER2 and IGF-1 signaling would improve treatment effect (25, 26). Herein, we included 679 HER2-positive BC patients and found that IGF-1 expression level was not associated with RFS, but patients with high IGF-1 level had significant better OS in the HER2-positive population. Others suggested that IGF-1R-mediated signaling pathways played a role in mediating *de novo* or acquired trastuzumab resistance (22, 23), probably due to a physical interaction between IGF-1R and HER2 as previously described (24). It was hypothesized that IGF-1R promoted proliferation and invasion through Forkhead box protein M1 and Src-focal adhesion kinase signaling (27). Cotargeting IGF-1R and HER2 by combined IGF-1R antibodies and trastuzumab resulted in cell growth inhibition (27). Further evaluation of paired IGF-1, IGF-1R, and IGFBPs should be carried out to better understand their effects on disease outcome.

IGF-1 was supposed to be a key hormone in the pathophysiology of MetS since it was implicated in the metabolism of glucose, carbohydrates and lipids. Nevertheless, the mechanism of this possible relationship is rather complicated, as multiple factors interact to control IGF-1 levels. It was well-established that IGF-1 level varied according to age, with an initially increase from birth to puberty, followed by a stable decline with age (28). Here we didn't observe an apparent correlation between IGF-1 and age (OR 1.04, 95% CI 0.53–2.05, *P* = 0.905), but IGF-1 expression was lower in post-menopausal patients compared to pre/peri-menopausal ones (OR 4.00, 95% CI 2.08–7.69, *P* < 0.001). Other identified impact factors of IGF-1 expression included height, alcohol consumption and parity (29). Previous studies found that a low circulating IGF-1 level was independently related to insulin resistance and hyperglycaemia (25, 26, 30), while a normal to high circulating IGF-1 level independently led to a reduced prevalence of MetS (31). As shown in our data, patients in the high IGF-1 group reported fewer MetS components than those in the low IGF-1 group, but the significance existed only in the univariate model (Univariate *P* = 0.033; MetS components 1–2 vs. 0: OR 0.70, 95% CI 0.40–1.25, *P* = 0.229; MetS components ≥ 3 vs. 0: OR 0.57, 95% CI 0.26–1.23, *P* = 0.153). In addition, other members of IGF-1 system, including IGFBP-3 (*P* < 0.001), insulin (*P* < 0.001), and C-peptide (*P* = 0.001) had similar distribution pattern as IGF-1 in the univariate model, while only IGFBP-3 was significantly associated with IGF-1 expression by multivariate analysis (OR 5.48, 95% CI 3.61–8.31, *P* < 0.001).

Our findings proposed that obesity status should be taken into consideration when featuring the impact of IGF-1 expression on the prognosis of BC patients. In non-overweight patients, high IGF-1 expression led to a better RFS and OS compared to low IGF-1 group. On the opposite, in overweight patients, high IGF-1 expression was independently associated with worse RFS in both univariate and multivariate model. Our findings are consistent with Duggan et al. who showed that increasing IGF-1 level was significantly associated with an approximate 2-fold greater increased risk of BC-specific mortality in participants with a BMI > 25 kg/m^2^, but not in lean women (14). We further investigated the relationship between HER2 targeted therapy efficacy and IGF-1 interacted with obesity (Figure S2), and found that only in patients with BMI <24.0 kg/m^2^ receiving adjuvant targeted therapy, those with higher expression of IGF-1 had a significant superior OS than those with lower IGF-1 (Log-rank *P* < 0.001). This suggested that IGF-1 might be related to targeted therapy treatment resistance in certain circumstances. Obesity is a broadly accepted risk factor for BC development (20, 32–34). The relative risk for premenopausal and postmenopausal BC was 1.16 and 1.12 with every 5 kg/m^2^ increase in BMI, as shown in a study of 282,137 participants (32). Obesity is also related to decreased overall and BC survival. A recent meta-analysis including 213,075 BC patients estimated a 41% and a 7% increased risk of all-cause mortality in obese and overweight patients, respectively, compared to their normal-weight matched controls (35). Similarly, obese or overweight women carried a 35 or 11% increased risk of BC-specific mortality than normal-weighted ones (35). Multiple molecular alterations, including insulin resistance, IGF pathway and inflammatory cytokines hyperactivation, are involved in obesity, which is able to modulate the biological behavior of BC cells and tumor microenvironment (36). Although the exact mechanism of the interaction between IGF-1 and overweight remains unclear, our data led to several possible hypotheses. One possible key factor was leptin, a cytokine secreted mainly by adipoctyes and closely related to body weight and body fat (37). Leptin could induce a 2 to 3-fold increase in liver or muscle-derived IGF-1 in aged calorie-restricted rodent model (38). Ecker et al. found that obese mice exhibited elevated levels of leptin and IGF-1 and showed an accelerated tumor recurrence compared with lean ones (*P* < 0.001) (39). Such synergistic effect of leptin and IGF-1 could possibly explain why overweight patients with high IGF-1 expression had worse diseases outcomes. Nevertheless, the protective role of circulating IGF-1 expression in lean patients hasn't been reported yet. It is possible that another feedback signaling pathway contributes in the process. When further subdividing the study population by molecular subtype, we found that only in lean HER2-overexpressed subtype was higher IGF-1 expression associated with improved OS. In addition, we also found that IGFBP-3 had no apparent influence on RFS or OS in HER2-positive breast cancer patients, either overweight or not (Figure S5). Further exploration and validation are warranted in preclinical models and in larger clinical population.

There are several limitations in this study. First, due to the retrospective design, potential selection biases might exist. Second, we failed to demonstrate the survival benefit of HER2-positive patients receiving targeted therapy compared with those without targeted therapy. One possible explanation was that patients weren't randomized to receive targeted therapy. Those with more aggressive tumor behavior were more likely to receive trastuzumab, thus diluting the efficiency of targeted therapy. Meanwhile, no enough events were reported since the patients enrolled had relatively superior disease outcomes, the follow-up wasn't long enough and should be continued. In addition, BMI ≥ 24.0 kg/m^2^ was employed to define central obesity according to previous studies (19–21), which we believed applicable as surrogate for Chinese women. To note, other IGF-1 cut-off values adopted to classify patient population other than median value of 160 ng/mL might lead to different results. For example, when using the mean IGF-1 value of 168 ng/mL, we found that IGF-1 >168 ng/mL was associated with significantly impaired RFS in overweight patients (HR 3.13, 95% CI 1.05–9.35, *P* = 0.031; Table S6), and significant better OS in non-overweight patients (HR 0.20, 95% CI 0.04–0.91, *P* = 0.021; Table S7), which was generally consistent with previous analyses with cutoff of 160 ng/mL. When further divided by molecular subtype, we found that IGF-1 > 168 ng/mL was associated with much worse RFS in overweight HER2-overexpressed subtype patients (HR 5.13, 95% CI 1.04–25.42, *P* = 0.026). Moreover, we found that with the increase of IGF-1 by quartile, the risk of disease recurrence tended to linearly increase in overweight patients (Table S6), but not in non-overweight patients. The optimal cutoff should be further explored. Furthermore, we only conducted IGF-1 measurements at diagnosis, while monitoring the dynamic change of IGF-1 during follow-up may be needed to better predict disease outcome or evaluate metabolic status, especially with certain treatment targeting the IGF axis.

## Conclusions

In conclusion, circulating IGF-1 expression in HER2-positive breast cancer patients was associated with menopausal status and IGFBP-3 level. IGF-1 and BMI had significant interaction in predicting RFS and OS in HER2-positive patients, with high IGF-1 protective for non-overweight ones, but deleterious for overweight ones, which warrants further clinical evaluation.

## Data Availability Statement

The datasets generated for this study are available on request to the corresponding author.

## Ethics Statement

This study involving human participants was reviewed and approved by the independent Ethical Committees of Ruijin Hospital, Shanghai Jiaotong University School of Medicine. All procedures performed in studies involving human participants were in accordance with the ethical standards of the institutional and/or national research committee and with the 1964 Helsinki declaration and its later amendments or comparable ethical standards. The patients/participants provided their written informed consent to participate in this study.

## Author Contributions

YT analyzed and interpreted the patient data and was a major contributor in writing the manuscript. JW, OH, JH, LZ, WC, and YL contributed to the data collection and acquisition. XC made substantial contributions to the conception of the work, analyzed, and interpreted the patient data. KS substantively revised the manuscript.

## Conflict of Interest

The authors declare that the research was conducted in the absence of any commercial or financial relationships that could be construed as a potential conflict of interest.
