# Supplementary material for: IGF-1 Interacted With Obesity in Prognosis Prediction in HER2-Positive Breast Cancer Patients
Source: Front Oncol. 2020 Apr 24;10:550. doi: 10.3389/fonc.2020.00550 (PMC7193870; doi:10.3389/fonc.2020.00550)
Supplement: Supplementary file 1 [file Table_1.docx]

**Supplementary Table S1. Distribution of MetS-related variables among recurrence-free and recurrent patients**

| **Variables** | | **Recurrence-free**  **N=627** | **Recurrent**  **N=52** | ***P***  **value** |
| --- | --- | --- | --- | --- |
| BMI, kg/m^2^ | | 23.06 (22.82, 23.31) | 22.74 (21.85, 23.64) | 0.477 |
| Overweight | | 222 (35.41) | 14 (26.92) | 0.217 |
| Elevated blood pressure | | 327 (52.15) | 21 (40.38) | 0.103 |
| Fasting glucose, mmol/L | | 5.38 (5.29, 5.46) | 5.36 (5.09, 5.62) | 0.899 |
| Elevated fasting glucose | | 154 (24.56) | 13 (25.00) | 0.944 |
| IGF-1, ng/mL | | 167.61 (162.89, 172.34) | 173.79 (154.59, 192.99) | 0.483 |
| High IGF-1 expression | | 317 (50.56) | 25 (48.08) | 0.731 |
| IGFBP-3, μg/mL* | | 3.04 (2.89, 3.19) | 2.65 (2.08, 3.22) | 0.167 |
| High IGFBP-3 expression | | 235 (50.32) | 16 (47.06) | 0.713 |
| IGF-1/ IGFBP-3 (×10^-3^)* | | 40.53 (39.47, 41.59) | 45.14 (39.69, 50.58) | **0.030** |
| Insulin, μIU/mL | | 9.29 (8.37, 10.20) | 9.64 (7.71, 11.57) | 0.830 |
| High insulin level | | 313 (49.92) | 27 (51.92) | 0.781 |
| C-peptide, μg/L | | 2.04 (1.98, 2.09) | 2.24 (1.99, 2.50) | **0.045** |
| High C-peptide level | | 313 (49.92) | 28 (53.85) | 0.586 |
| Triglycerides, mmol/L | | 1.30 (1.25,1.36) | 1.25 (1.10, 1.40) | 0.601 |
| Elevated triglycerides | | 131 (20.89) | 9 (17.31) | 0.539 |
| Total cholesterol, mmol/L | | 4.91 (4.84, 4.99) | 4.89 (4.64, 5.14) | 0.879 |
| HDL-C, mmol/L | | 1.37 (1.34,1.40) | 1.39 (1.29, 1.49) | 0.627 |
| Reduced HDL-C |  | 279 (44.50) | 25 (38.08) | 0.618 |
| LDL-C, mmol/L | | 3.02 (2.95, 3.08) | 3.04 (2.85, 3.23) | 0.839 |
| MetS at diagnosis |  | 196 (31.26) | 13 (25.00) | 0.347 |
| No. of MetS components | | 1.78 (1.67, 1.89) | 1.60 (1.23, 1.96) | 0.371 |

Numeric data presented as mean (95% confidence interval), categorical data presented as number (percentage).

*: There were 178 missing values for IGFBP-3 or IGF-1/ IGFBP-3 ratio, 160 in the recurrence-free group and 18 in the recurrent group.

Abbreviations: MetS, metabolic syndrome; BMI, body mass index; IGF-1, insulin-like growth factor 1; IGFBP-3, insulin-like growth factor binding protein-3; HDL-C, high-density lipoprotein-cholesterol; LDL-C, low-density lipoprotein-cholesterol; No., number.

**Supplementary Table S2. Univariate analysis of prognostic factors affecting RFS**

| Factors | Whole patients | Non-overweight | Overweight |
| --- | --- | --- | --- |
|  | *P* value | *P* value | *P* value |
| BMI, kg/m^2^ | 0.215 |  |  |
| Age at diagnosis, years | 0.149 | 0.874 | **0.003** |
| Menopausal status | 0.702 | 0.391 | 0.053 |
| Histologic type | 0.225 | 0.331 | 0.423 |
| Histological grade | 0.638 | 0.565 | 0.885 |
| Tumor size, cm | **0.014** | 0.144 | **0.020** |
| Node status | **<0.001** | **<0.001** | 0.405 |
| ER status | **0.002** | **<0.001** | 0.862 |
| PR status | **0.009** | **0.001** | 0.522 |
| Ki67, % | 0.164 | 0.061 | 0.701 |
| Molecular subtype | **0.002** | **<0.001** | 0.862 |
| IGF-1, ng/mL | 0.620 | **0.049** | **0.038** |
| IGFBP-3, μg/mL* | 0.546 | 0.313 | 0.714 |
| IGF-1/ IGFBP-3 (×10^-3^)* | 0.211 | 0.953 | 0.051 |
| MetS at diagnosis | 0.308 | 0.292 | 0.522 |
| Chemotherapy | 0.746 | 0.691 | 0.209 |
| Radiotherapy | **0.025** | **0.018** | 0.555 |
| Targeted therapy | 0.402 | 0.419 | 0.735 |
| Endocrine therapy | **0.003** | **<0.001** | 0.889 |

*: There were 178 missing values for IGFBP-3 or IGF-1/ IGFBP-3 ratio.

Abbreviations: RFS, recurrence-free survival; HER2, human epidermal growth factor receptor-2; ER, estrogen receptor; PR, progesterone receptor; IGF-1, insulin-like growth factor 1; MetS, metabolic syndrome; BMI, body mass index.

**Supplementary Table S3. Multivariate analysis of prognostic factors affecting RFS and OS in HER2-positive breast cancer patients**

| Factors | RFS | | |  | OS | | |
| --- | --- | --- | --- | --- | --- | --- | --- |
|  | HR | 95% CI | *P* |  | HR | 95% CI | *P* |
| Age, years ($\geq$50 *vs* $<$50) | 0.54 | 0.31-0.94 | **0.028** |  | 0.33 | 0.12-0.93 | **0.036** |
| Tumor size, cm ($\geq$2.0 *vs* $<$2.0) | 1.72 | 0.94-3.17 | 0.079 |  | 2.83 | 0.78-10.25 | 0.113 |
| Node status (Positive *vs* Negative) | 3.30 | 1.86-5.84 | **<0.001** |  | 6.78 | 1.91-24.03 | **0.003** |
| ER status (Positive *vs* Negative) | 0.37 | 0.19-0.70 | **0.002** |  | 1.39 | 0.37-5.23 | 0.624 |
| PR status (Positive *vs* Negative) | 0.27 | 0.11-0.64 | **<0.001** |  | 0.31 | 0.06-1.61 | 0.164 |
| IGF-1, ng/mL ($\geq$160 *vs* $<$160) | 0.85 | 0.49-1.48 | 0.571 |  | 0.26 | 0.08-0.82 | **0.022** |

Abbreviations: RFS, recurrence-free survival; OS, overall survival; HER2, human epidermal growth factor receptor-2; HR, hazard ratio; CI, confidence interval; ER, estrogen receptor; PR, progesterone receptor; IGF-1, insulin-like growth factor 1.

**Supplementary Table S4. Univariate analysis of prognostic factors affecting OS**

| Factors | Whole patients | Non-overweight | Overweight |
| --- | --- | --- | --- |
|  | *P* value | *P* value | *P* value |
| BMI, kg/m^2^ | 0.227 |  |  |
| Age at diagnosis, years | **0.038** | 0.522 | **0.001** |
| Menopausal status | 0.690 | 0.369 | **0.015** |
| Histologic type | 0.853 | 0.744 | 0.732 |
| Histological grade | 0.565 | 0.167 | 0.190 |
| Tumor size, cm | **0.045** | **0.040** | 0.688 |
| Node status | **0.001** | **<0.001** | 0.842 |
| ER status | 0.747 | 0.435 | 0.413 |
| PR status | 0.525 | 0.143 | 0.123 |
| Ki67, % | 0.251 | 0.205 | 0.899 |
| Molecular subtype | 0.747 | 0.435 | 0.413 |
| IGF-1, ng/mL | **0.041** | **0.005** | 0.438 |
| IGFBP-3, μg/mL* | 0.800 | 0.837 | 0.317 |
| IGF-1/ IGFBP-3 (×10^-3^)* | 0.271 | 0.080 | 0.259 |
| MetS at diagnosis | 0.305 | 0.799 | 0.231 |
| Chemotherapy | 0.944 | 0.643 | 0.319 |
| Radiotherapy | 0.975 | 0.517 | 0.126 |
| Targeted therapy | 0.149 | 0.115 | 0.843 |
| Endocrine therapy | 0.469 | 0.182 | 0.333 |

*: There were 178 missing values for IGFBP-3 or IGF-1/ IGFBP-3 ratio.

Abbreviations: OS, overall survival; HER2, human epidermal growth factor receptor-2; ER, estrogen receptor; PR, progesterone receptor; IGF-1, insulin-like growth factor 1; MetS, metabolic syndrome; BMI, body mass index. **Supplementary Table S5. Multivariate analysis of prognostic factors affecting OS in HER2-positive breast cancer patients by BMI status**

| Factors | Non-overweight | | |  | Overweight | | |
| --- | --- | --- | --- | --- | --- | --- | --- |
|  | HR | 95% CI | *P* |  | HR | 95% CI | *P* |
| Age, years ($\geq$50 *vs* $<$50) | 0.15 | 0.03-0.71 | **0.016** |  | 0.00 | 0.00-∞ | 0.959 |
| Menstruation (PostM vs Pre/PeriM) | 3.23 | 0.63-16.48 | 0.158 |  | 0.00 | 0.00-∞ | 0.961 |
| Tumor size, cm ($\geq$2.0 *vs* $<$2.0) | 2.97 | 0.63-13.96 | 0.169 |  | 1.74 | 0.12-24.46 | 0.681 |
| Node status (Positive *vs* Negative) | 19.57 | 2.53-151.64 | **0.004** |  | 0.85 | 0.07-11.08 | 0.854 |
| IGF-1, ng/mL ($\geq160 vs<160$) | 0.15 | 0.03-0.71 | **0.016** |  | 2.13 | 0.17-25.00 | 0.554 |

Abbreviations: OS, overall survival; HER2, human epidermal growth factor receptor-2; BMI, body mass index; HR, hazard ratio; CI, confidence interval; PostM, post-menopausal; Pre/PeriM, pre/peri-menopausal; IGF-1, insulin-like growth factor 1.

**Supplementary Table S6. Impact of IGF-1 on RFS among HER2-positive patients by molecular subtype and BMI status.**

| Subgroup | Hazard ratio (95% confidence interval) | | |
| --- | --- | --- | --- |
|  | Whole patients | Luminal B HER2+ | HER2-overexpressed |
| Whole population |  |  |  |
| Mean (High *vs* Low) | 0.94 (0.54-1.63) | 1.16 (0.37-3.59) | 0.92 (0.49-1.74) |
| Median (High *vs* Low) | 0.87 (0.51-1.50) | 1.26 (0.40-3.96) | 0.80 (0.43-1.50) |
| Quartile (Q2 *vs* Q1) | 1.18 (0.55-2.50) | 0.74 (0.12-4.48) | 1.36 (0.59-3.14) |
| (Q3 *vs* Q1) | 0.69 (0.30-1.61) | 1.10 (0.22-5.47) | 0.58 (0.21-1.59) |
| (Q4 *vs* Q1) | 1.27 (0.61-2.64) | 1.22 (0.27-5.47) | 1.40 (0.60-3.23) |
| Non-overweight |  |  |  |
| Mean (High *vs* Low) | 0.57 (0.29-1.11) | 0.93 (0.19-4.59) | 0.56 (0.27-1.19) |
| Median (High *vs* Low) | 0.53 (0.27-1.01) | 0.72 (0.15-3.57) | 0.53 (0.26-1.08) |
| Quartile (Q2 *vs* Q1) | 1.04 (0.46-2.36) | 0.52 (0.05-5.70) | 1.27 (0.53-3.06) |
| (Q3 *vs* Q1) | 0.37 (0.13-1.04) | 0.47 (0.04-5.15) | 0.35 (0.11-1.12) |
| (Q4 *vs* Q1) | 0.73 (0.31-1.68) | 0.64 (0.09-4.56) | 0.91 (0.36-2.31) |
| Overweight |  |  |  |
| Mean (High *vs* Low) | **3.13 (1.05-9.35)** | 1.81 (0.36-8.96) | **5.13 (1.04-25.42)** |
| Median (High *vs* Low) | **3.20 (1.00-10.21)** | 2.78 (0.51-15.21) | 3.68 (0.74-18.23) |
| Quartile (Q2 *vs* Q1) | 3.17 (0.33-30.53) | 1.30 (0.08-21.25) | ∞ (0.00-∞) |
| (Q3 *vs* Q1) | 5.29 (0.59-47.35) | 3.03 (0.27-33.86) | ∞ (0.00-∞) |
| (Q4 *vs* Q1) | **8.72 (1.05-72.43)** | 3.89 (0.35-43.23) | ∞ (0.00-∞) |

Abbreviations: IGF-1, insulin-like growth factor 1; RFS, recurrence-free survival; HER2, human epidermal growth factor receptor-2; BMI, body mass index.

**Supplementary Table S7. Impact of IGF-1 on OS among HER2-positive patients by molecular subtype and BMI status.**

| Subgroup | Hazard ratio (95% confidence interval) | | |
| --- | --- | --- | --- |
|  | Whole patients | Luminal B HER2+ | HER2-overexpressed |
| Whole population |  |  |  |
| Mean (High *vs* Low) | 0.44 (0.14-1.37) | 0.55 (0.10-3.00) | 0.37 (0.08-1.77) |
| Median (High *vs* Low) | 0.32 (0.10-1.01) | 0.42 (0.08-2.30) | 0.26 (0.05-1.27) |
| Quartile (Q2 *vs* Q1) | 1.37 (0.42-4.51) | 1.11 (0.16-7.91) | 1.58 (0.35-7.07) |
| (Q3 *vs* Q1) | 0.19 (0.02-1.61) | 0.00 (0.00-∞) | 0.30 (0.03-2.88) |
| (Q4 *vs* Q1) | 0.61 (0.15-2.53) | 0.88 (0.12-6.25) | 0.38 (0.04-3.64) |
| Non-overweight |  |  |  |
| Mean (High *vs* Low) | **0.20 (0.04-0.91)** | 0.26 (0.03-2.57) | 0.17 (0.02-1.38) |
| Median (High *vs* Low) | **0.15 (0.03-0.68)** | 0.20 (0.02-1.98) | 0.12 (0.02-1.01) |
| Quartile (Q2 *vs* Q1) | 1.19 (0.34-4.12) | 0.53 (0.05-5.91) | 1.80 (0.40-8.21) |
| (Q3 *vs* Q1) | 0.00 (0.00-∞) | 0.00 (0.00-∞) | 0.00 (0.00-∞) |
| (Q4 *vs* Q1) | 0.34 (0.07-1.74) | 0.28 (0.03-3.18) | 0.40 (0.04-3.82) |
| Overweight |  |  |  |
| Mean (High *vs* Low) | 3.32 (0.30-36.69) | 1.81 (0.11-28.93) | 86.37 (0.00-∞) |
| Median (High *vs* Low) | 2.51 (0.23-27.63) | 1.27 (0.08-20.31) | 65.29 (0.00-∞) |
| Quartile (Q2 *vs* Q1) | ∞ (0.00-∞) | ∞ (0.00-∞) | 1.00 (0.00-∞) |
| (Q3 *vs* Q1) | ∞ (0.00-∞) | 1.00 (0.00-∞) | 434.45 (0.00-∞) |
| (Q4 *vs* Q1) | ∞ (0.00-∞) | ∞ (0.00-∞) | 1.00 (0.00-∞) |

Abbreviations: IGF-1, insulin-like growth factor 1; OS, overall survival; HER2, human epidermal growth factor receptor-2; BMI, body mass index.
