# Supplementary material for: IGF-1 Interacted With Obesity in Prognosis Prediction in HER2-Positive Breast Cancer Patients
Source: Front Oncol. 2020 Apr 24;10:550. doi: 10.3389/fonc.2020.00550 (PMC7193870; doi:10.3389/fonc.2020.00550)
Supplement: Supplementary file 2 [file Data_Sheet_1.docx]

**Supplementary Figure Legends**

**Supplementary Figure S1. Impact of IGF-1 on RFS and its interaction with BMI by molecular subtype.** (A) For Luminal B HER2+ patients with BMI<24.0 kg/m^2^, those with higher expression of IGF-1 had similar RFS to those with lower IGF-1 (Log-rank *P*=0.687). (B) For Luminal B HER2+ patients with BMI≥24.0 kg/m^2^, those with higher expression of IGF-1 had similar RFS to those with lower IGF-1 (Log-rank *P*=0.219). (C) For HER2-overexpressed patients with BMI<24.0 kg/m^2^, those with higher expression of IGF-1 had similar RFS to those with lower IGF-1 (Log-rank *P*=0.074). (D) For HER2-overexpressed patients with BMI≥24.0 kg/m^2^, those with higher expression of IGF-1 had similar RFS to those with lower IGF-1 (Log-rank *P*=0.087).

Abbreviations: IGF-1, insulin-like growth factor-1; RFS, recurrence-free survival; BMI, body mass index; HER2, human epidermal growth factor receptor 2; No, number.

**Supplementary Figure S2. Impact of IGF-1 on prognosis and its interaction with BMI by use of adjuvant targeted therapy.** (A) For patients with BMI<24.0 kg/m^2^ and received adjuvant targeted therapy, those with higher expression of IGF-1 had similar RFS to those with lower IGF-1 (Log-rank *P*=0.053). (B) For patients with BMI<24.0 kg/m^2^ and received adjuvant targeted therapy, those with higher expression of IGF-1 had significant superior OS than those with lower IGF-1 (Log-rank *P*<0.001). (C) For patients with BMI<24.0 kg/m^2^ and didn’t receive adjuvant targeted therapy, those with higher expression of IGF-1 had similar RFS to those with lower IGF-1 (Log-rank *P*=0.584). (D) For patients with BMI<24.0 kg/m^2^ and didn’t receive adjuvant targeted therapy, those with higher expression of IGF-1 had similar OS to those with lower IGF-1 (Log-rank *P*=0.686). (E) For patients with BMI≥24.0 kg/m^2^ and received adjuvant targeted therapy, those with higher expression of IGF-1 had similar RFS to those with lower IGF-1 (Log-rank *P*=0.125). (F) For patients with BMI≥24.0 kg/m^2^ and received adjuvant targeted therapy, those with higher expression of IGF-1 had similar OS to those with lower IGF-1 (Log-rank *P*=0.946). (G) For patients with BMI≥24.0 kg/m^2^ and didn’t receive adjuvant targeted therapy, those with higher expression of IGF-1 had similar RFS to those with lower IGF-1 (Log-rank *P*=0.116). (H) For patients with BMI≥24.0 kg/m^2^ and didn’t receive adjuvant targeted therapy, those with higher expression of IGF-1 had similar OS to those with lower IGF-1 (Log-rank *P*=0.292).

Abbreviations: IGF-1, insulin-like growth factor-1; RFS, recurrence-free survival; OS, overall survival; BMI, body mass index; No, number.

**Supplementary Figure S3. Forest plots and interaction analysis for OS in HER2-positive breast cancer patients with different level of IGF-1.**

Abbreviations: OS, overall survival; HER2, human epidermal growth factor receptor 2; IGF-1, insulin-like growth factor-1; HR, hazard ratio; CI, confidence interval; BMI, body mass index; IDC, invasive ductal carcinoma; ER, estrogen receptor; HR, hormonal receptor; IGFBP-3, insulin-like growth factor binding protein-3.

**Supplementary Figure S4. Impact of IGF-1 on OS and its interaction with BMI by molecular subtype.** (A) For Luminal B HER2+ patients with BMI<24.0 kg/m^2^, those with higher expression of IGF-1 had similar OS to those with lower IGF-1 (Log-rank *P*=0.129). (B) For Luminal B HER2+ patients with BMI≥24.0 kg/m^2^, those with higher expression of IGF-1 had similar OS to those with lower IGF-1 (Log-rank *P*=0.865). (C) For HER2-overexpressed patients with BMI<24.0 kg/m^2^, those with higher expression of IGF-1 had significantly improved OS than those with lower IGF-1 (Log-rank *P*=0.020). (D) For HER2-overexpressed patients with BMI≥24.0 kg/m^2^, those with higher expression of IGF-1 had similar OS to those with lower IGF-1 (Log-rank *P*=0.317).

Abbreviations: IGF-1, insulin-like growth factor-1; OS, overall survival; BMI, body mass index; HER2, human epidermal growth factor receptor 2; No, number.

**Supplementary Figure S5. Impact of IGFBP-3 on survival and its interaction with BMI.** (A) IGFBP-3 expression was not associated with RFS in the whole population (Log-rank *P*=0.546). (B) For patients with BMI<24.0 kg/m^2^, those with higher expression of IGFBP-3 had similar RFS to those with lower IGFBP-3 (Log-rank *P*=0.690). (C) For patients with BMI≥24.0 kg/m^2^, those with higher expression of IGFBP-3 had similar RFS to those with lower IGFBP-3 (Log-rank *P*=0.725). (D) IGFBP-3 expression was not associated with OS in the whole population (Log-rank *P*=0.801). (E) For patients with BMI<24.0 kg/m^2^, those with higher expression of IGFBP-3 had similar OS to those with lower IGFBP-3 (Log-rank *P*=0.654). (F) For patients with BMI≥24.0 kg/m^2^, those with higher expression of IGFBP-3 had similar OS to those with lower IGFBP-3 (Log-rank *P*=0.228).

Abbreviations: IGFBP-3, insulin-like growth factor binding protein-3; BMI, body mass index; RFS, recurrence-free survival; No, number.
