# Supplementary material for: IGF-1 Interacted With Obesity in Prognosis Prediction in HER2-Positive Breast Cancer Patients
Source: Front Oncol. 2020 Apr 24;10:550. doi: 10.3389/fonc.2020.00550 (PMC7193870; doi:10.3389/fonc.2020.00550)
Supplement: Supplementary file 3 [file Image_1.pdf]

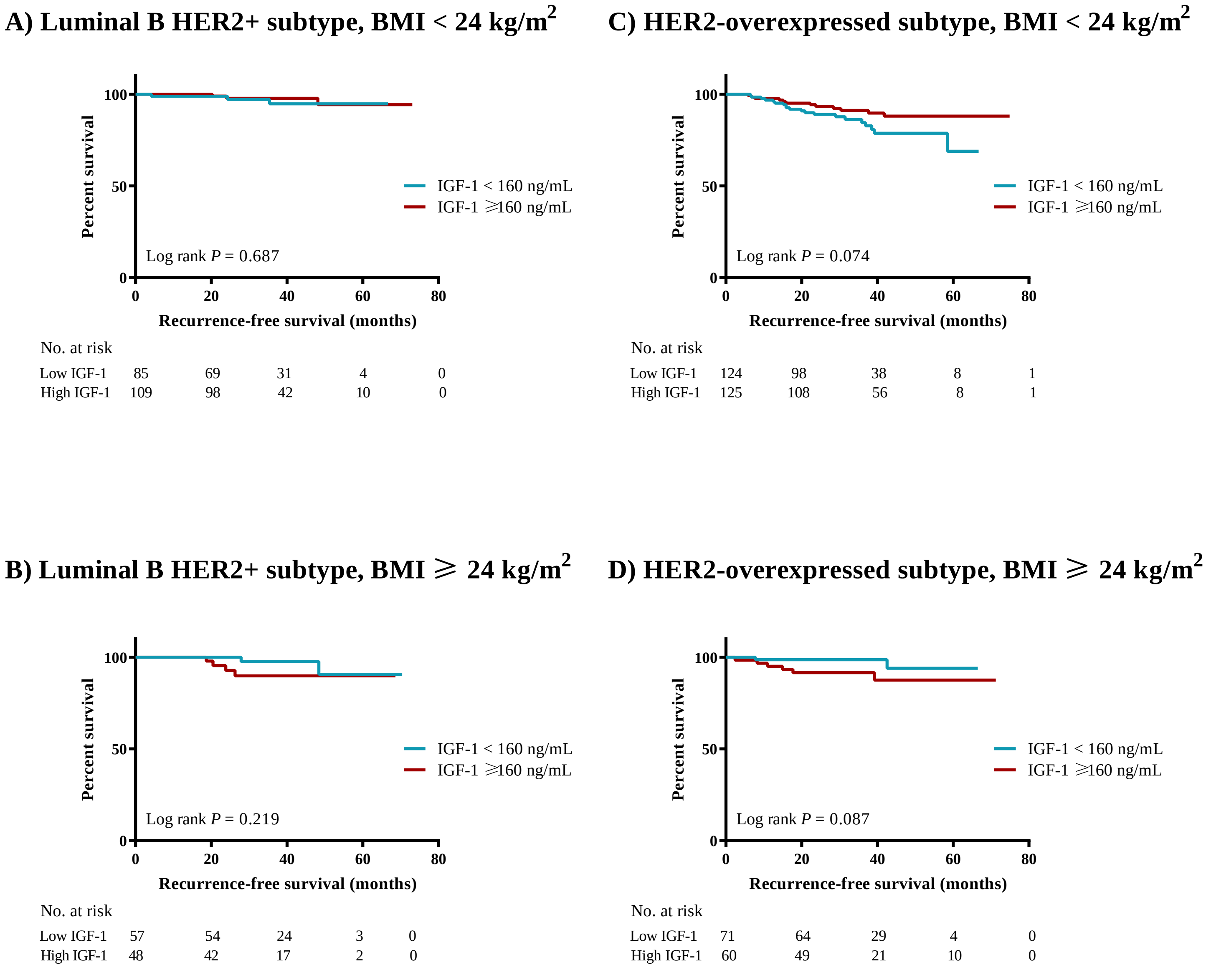
A) Luminal B HER2+ subtype, BMI < 24 kg/m2
Percent survival
100
50
0
IGF-1 < 160 ng/mL
IGF-1 ≥160 ng/mL
Log rank P = 0.687
0 20 40 60 80
Recurrence-free survival (months)
No. at risk
Low IGF-1 85 69 31 4 0
High IGF-1 109 98 42 10 0
C) HER2-overexpressed subtype, BMI < 24 kg/m2
Percent survival
100
50
0
IGF-1 < 160 ng/mL
IGF-1 ≥160 ng/mL
Log rank P = 0.074
0 20 40 60 80
Recurrence-free survival (months)
No. at risk
Low IGF-1 124 98 38 8 1
High IGF-1 125 108 56 8 1
B) Luminal B HER2+ subtype, BMI ≥ 24 kg/m2
Percent survival
100
50
0
IGF-1 < 160 ng/mL
IGF-1 ≥160 ng/mL
Log rank P = 0.219
0 20 40 60 80
Recurrence-free survival (months)
No. at risk
Low IGF-1 57 54 24 3 0
High IGF-1 48 42 17 2 0
D) HER2-overexpressed subtype, BMI ≥ 24 kg/m2
Percent survival
100
50
0
IGF-1 < 160 ng/mL
IGF-1 ≥160 ng/mL
Log rank P = 0.087
0 20 40 60 80
Recurrence-free survival (months)
No. at risk
Low IGF-1 71 64 29 4 0
High IGF-1 60 49 21 10 0
