# Supplementary material for: IGF-1 Interacted With Obesity in Prognosis Prediction in HER2-Positive Breast Cancer Patients
Source: Front Oncol. 2020 Apr 24;10:550. doi: 10.3389/fonc.2020.00550 (PMC7193870; doi:10.3389/fonc.2020.00550)
Supplement: Supplementary file 4 [file Image_2.pdf]

A) Non-overweight, targeted therapy, RFS

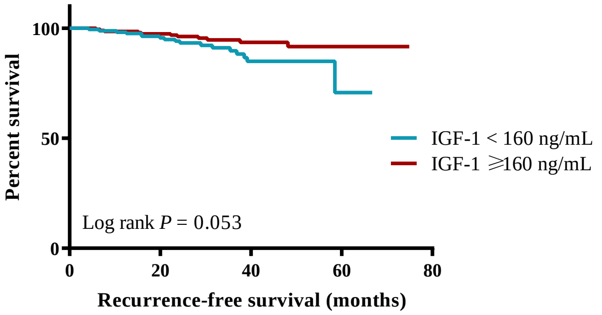

No. at risk

Low IGF-1	164	131	49	6	0
High IGF-1	196	170	74	14	0

B) Non-overweight, targeted therapy, OS

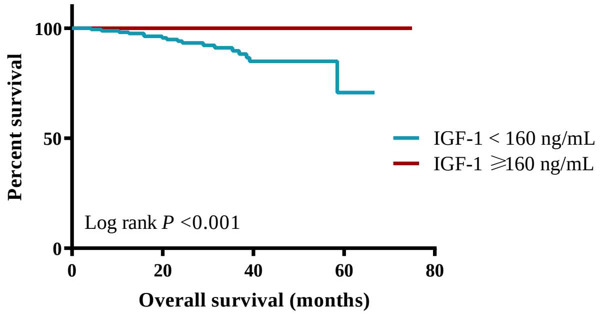

No. at risk

Low IGF-1	164	131	49	6	0
High IGF-1	196	178	77	15	0

C) Non-overweight, no targeted therapy, RFS

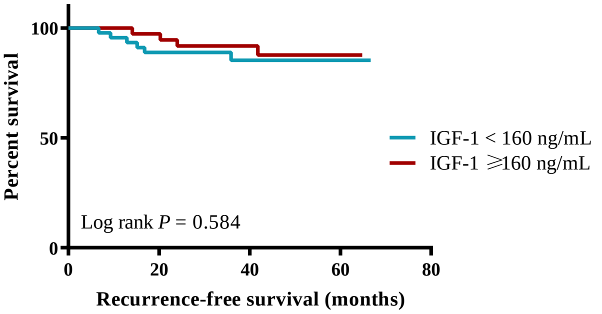

No. at risk

Low IGF-1	45	37	21	7	0
High IGF-1	38	37	25	5	0

D) Non-overweight, no targeted therapy, OS

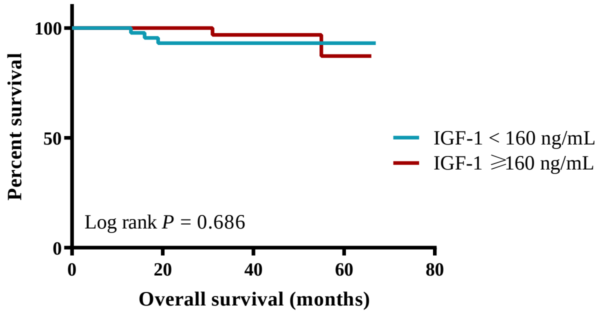

No. at risk

Low IGF-1	45	39	22	7	0
High IGF-1	38	38	26	6	0

E) Overweight, targeted therapy, RFS

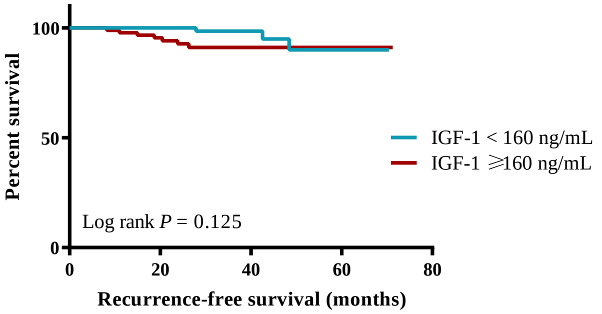

No. at risk

Low IGF-1	94	86	40	5	0
High IGF-1	90	75	28	9	0

F) Overweight, targeted therapy, OS

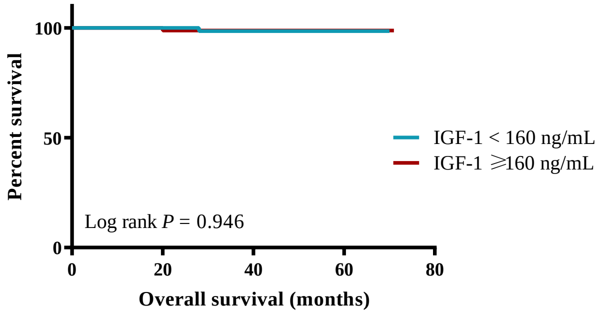

No. at risk

Low IGF-1	94	86	40	7	0
High IGF-1	90	76	33	9	0

G) Overweight, no targeted therapy, RFS

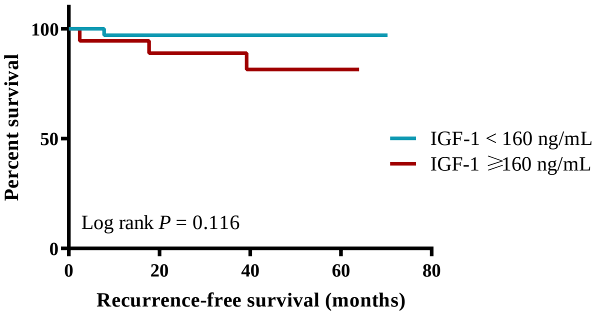

No. at risk

Low IGF-1	34	32	15	4	0
High IGF-1	18	17	12	5	0

H) Overweight, no targeted therapy, OS

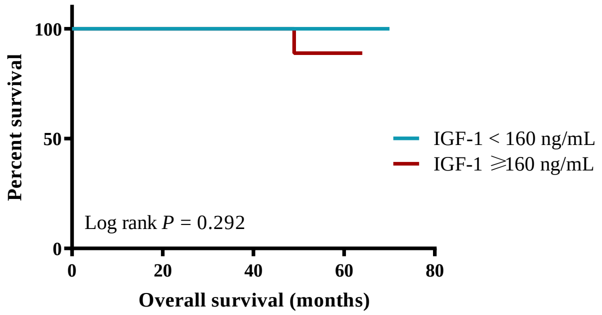

No. at risk

Low IGF-1	34	32	15	6	0
High IGF-1	18	18	14	5	0
