# Supplementary figures and images for: IGF-1 Interacted With Obesity in Prognosis Prediction in HER2-Positive Breast Cancer Patients
Source: Front Oncol. 2020 Apr 24;10:550. doi: 10.3389/fonc.2020.00550 (PMC7193870; doi:10.3389/fonc.2020.00550)

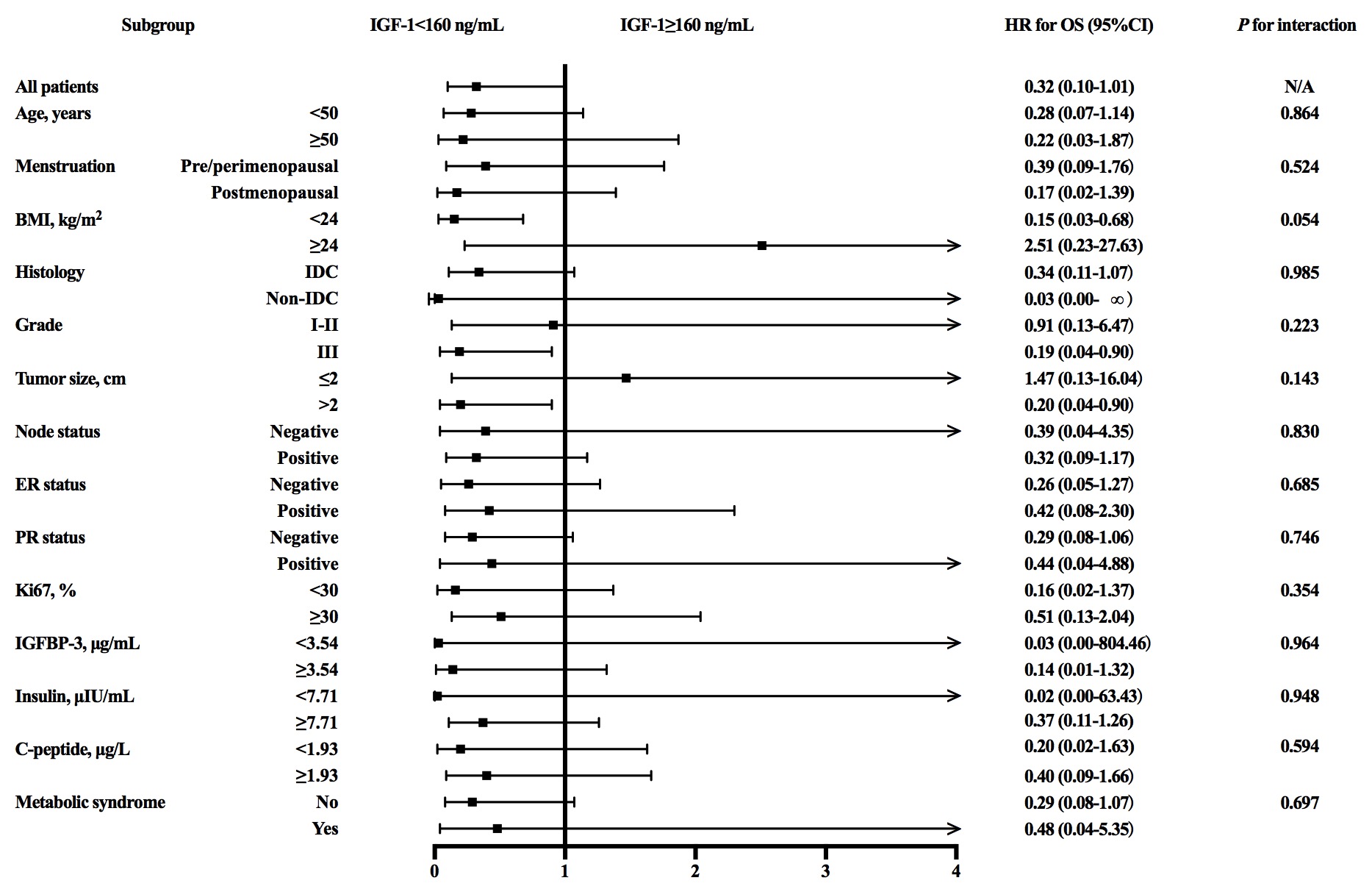

Supplement: Supplementary file 5 [file Image_3.jpeg]
