# Supplementary material for: IGF-1 Interacted With Obesity in Prognosis Prediction in HER2-Positive Breast Cancer Patients
Source: Front Oncol. 2020 Apr 24;10:550. doi: 10.3389/fonc.2020.00550 (PMC7193870; doi:10.3389/fonc.2020.00550)
Supplement: Supplementary file 6 [file Image_4.pdf]

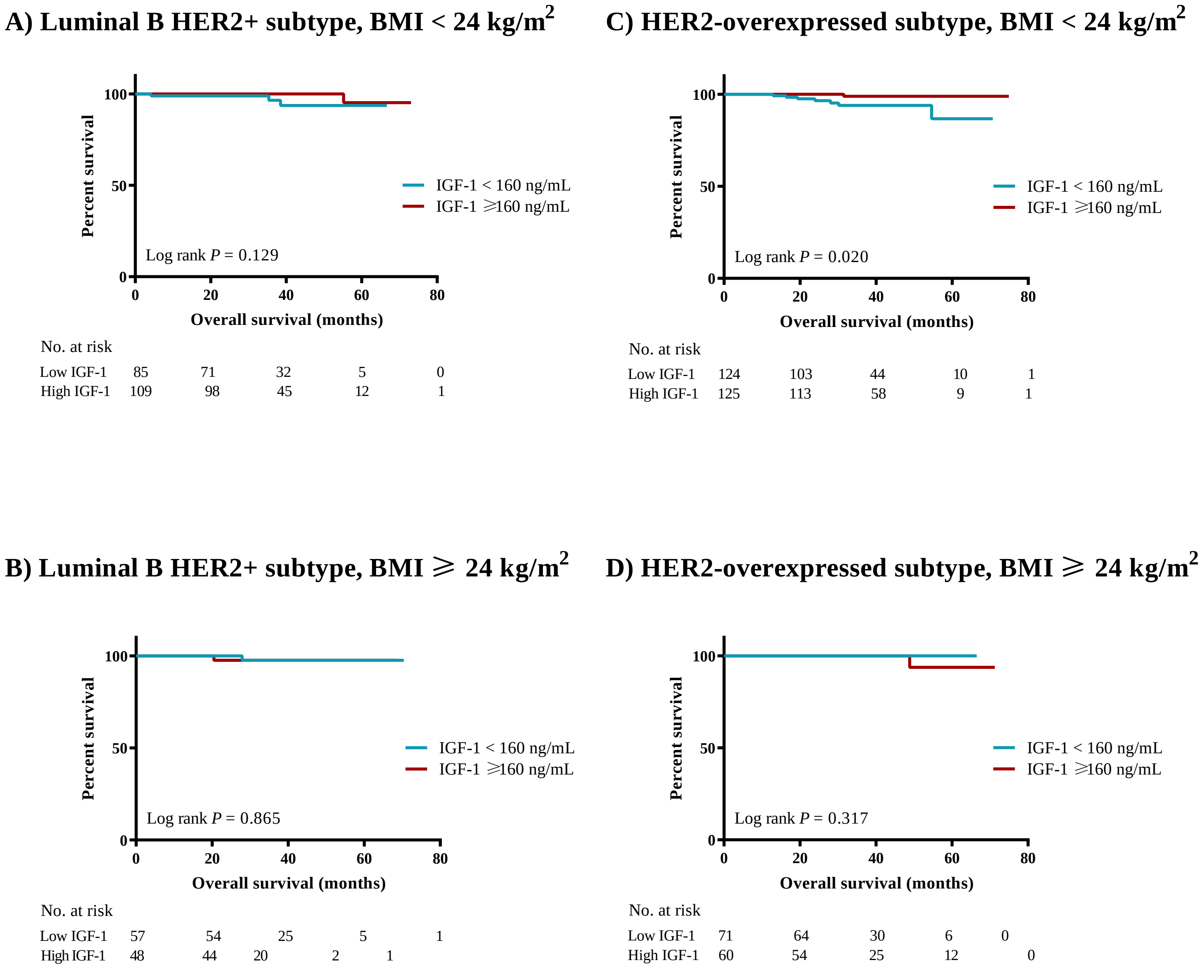
A) Luminal B HER2+ subtype, BMI < 24 kg/m2
Percent survival
100
50
0
0 20 40 60 80
Overall survival (months)
IGF-1 < 160 ng/mL
IGF-1 ⩾160 ng/mL
Log rank P = 0.129
No. at risk
Low IGF-1 85 71 32 5 0
High IGF-1 109 98 45 12 1
C) HER2-overexpressed subtype, BMI < 24 kg/m2
Percent survival
100
50
0
0 20 40 60 80
Overall survival (months)
IGF-1 < 160 ng/mL
IGF-1 ⩾160 ng/mL
Log rank P = 0.020
No. at risk
Low IGF-1 124 103 44 10 1
High IGF-1 125 113 58 9 1
B) Luminal B HER2+ subtype, BMI ⩾ 24 kg/m2
Percent survival
100
50
0
0 20 40 60 80
Overall survival (months)
IGF-1 < 160 ng/mL
IGF-1 ⩾160 ng/mL
Log rank P = 0.865
No. at risk
Low IGF-1 57 54 25 5 1
High IGF-1 48 44 20 2 1
D) HER2-overexpressed subtype, BMI ⩾ 24 kg/m2
Percent survival
100
50
0
0 20 40 60 80
Overall survival (months)
IGF-1 < 160 ng/mL
IGF-1 ⩾160 ng/mL
Log rank P = 0.317
No. at risk
Low IGF-1 71 64 30 6 0
High IGF-1 60 54 25 12 0
